# Variability in neural networks

**DOI:** 10.7554/eLife.34153

**Published:** 2018-01-18

**Authors:** Daniel R Kick, David J Schulz

**Affiliations:** 1 Division of Biological SciencesUniversity of Missouri-ColumbiaColumbiaUnited States

**Keywords:** leech heart system, central pattern generator, motor systems, pattern variability, physiology, Other

## Abstract

Experiments on neurons in the heart system of the leech reveal why rhythmic behaviors differ between individuals.

**Related research article** Wenning A, Norris B, Gunay C, Kueh D, Calabrese RL. 2018. Output variability across animals and levels in a motor system. *eLife* **7**:e31123. doi: 10.7554/eLife.31123

Many of our everyday behaviors, such as walking, breathing and chewing, are regulated by a network of neurons called a central pattern generator. Although these behaviors follow a similar pattern (we recognize walking when we see it), they can vary between individuals (everyone has a specific gait). Such differences can be found in both the building blocks and the activity of these networks (Swensen and Bean, 2005; Schulz et al., 2006; Bellardita and Kiehn, 2015; Pulver et al., 2015), and also between cells of the same cell type (Ransdell et al., 2013). Hence, what is often taken for granted as a ‘simple’ network or behavior in fact represents a substantial amount of variability and complexity across individuals.

All central pattern generators (CPGs) share common features, such as a pacemaker system that sets the rhythm, and the connections among the neurons that set the activity pattern of the network (Figure 1A). This CPG pattern is then transferred onto the motor neurons, which stimulate the muscles to produce the movements associated with the behavior. However, it is still unclear which components of the network contribute to individual differences, or if variations in one component could affect the variability of another.

**Figure 1.**
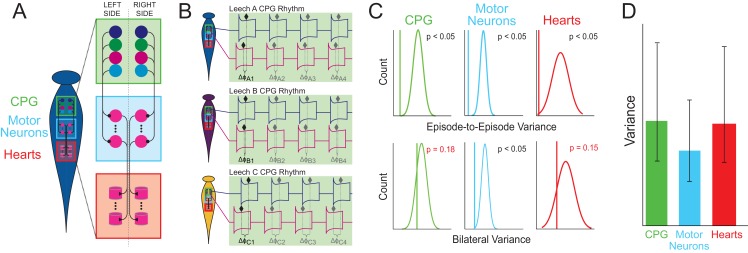
Schematic detailing the different components and activities within the leech heart system. (**A**) The heart system of the leech consists of two symmetrical tubes that run along the left and right side of the body. Each tube has components at three levels: the central pattern generator (CPG) interneurons (green box), which connect to the motor neurons (blue box), which in turn stimulate the heart muscles (red box). (**B**) Wenning et al. measured various phase differences in different leeches: for example, the phase difference *Δφ*_A1_ is the first measurement of the delay between the activation of two particular neurons in the CPG in leech A, and *Δφ*_C4_ is the fourth measurement of the delay between the activation of these neurons in leech C. Measuring how particular phase differences change over time allows the episode-to-episode variance to be determined, and comparing results for the left and right sides allowed the bilateral variance to be determined. (**C**) Wenning et al. compared their results (vertical lines) for episode-to-episode variance (top) and bilateral variance (bottom) with theoretical predictions for a randomized population (curves). Because the measured episode-to-episode variance was significantly different from the population variance (top), there must be physiological constraints on the episode-to-episode variability at all three levels. The measured bilateral variance was also significantly different from the population variance for motor neurons, but not for the CPG and the heart muscle component, which suggests that the two sides of a single animal are no more similar to one another than two randomly chosen individuals. (**D**) Comparisons across all three levels revealed that the motor neurons have lower variance than the CPG or heart muscle components. Error bars reflect 95% confidence intervals.

Now, in eLife, Angela Wenning, Ronald Calabrese and colleagues at Emory University, California State University San Marcos and Georgia Gwinnett College report how the timing of the activity of each CPG component can be used to measure variability (Wenning et al., 2018).

Building on previous research, Wenning at al. used the leech as a model system (Norris et al., 2011; Wenning et al., 2014). Leeches have two hearts, which are formed of tubes that run along the entire length of the body, one on each side of the animal. Moreover, these two hearts beat with different patterns. Driven by the CPG components, the tube on one side constricts and relaxes back-to-front, while the other side contracts all at once. Every few minutes, the tubes swap patterns.

To coordinate the pumping rhythm, the relative timing of neurons firing within a component (for example, between motor neurons at the front and the back), and also between components (for example, from the CPG pattern to the motor neurons), needs to be maintained. This timing can be measured as the difference in phase (which is the delay between the activation of one neuron and another; Figure 1B). Using these measurements, Wenning et al. set out to determine what causes the variability in network output between individual leeches.

The results revealed that the activity of CPG neurons, motor neurons and muscles varied greatly between leeches, but less so within each leech. In individual leeches, the phase difference at a given level remained fairly stable (Figure 1B). In other words, when one animal decides how it will generate its heartbeat, it sticks with it with great precision cycle after cycle. This does, however, vary from animal to animal.

To investigate this further, Wenning et al. compared the repetition-to-repetition variance measured in the leeches to a theoretical population variance produced by random pairings of the data (Figure 1C; top). This showed that the measured variance was significantly lower than the variance from a randomized population. This means that variations on a given side of an animal are small and the output is consistent. When Wenning et al. then compared the components of each level on both sides of the same animal, they discovered the two sides were no more similar to each other than to any other animal (Figure 1C; bottom). Therefore, although genetically identical, the CPGs on the left and right side of one leech can generate outputs that are as different from each other as the outputs from CPGs in different leeches.

In a feed-forward network such as the CPG system, one might expect any variability to increase at each level, or for there to be mechanisms in place to constrain such increases. However, Wenning et al. found neither of these results, and instead showed that the motor-neuron patterns varied less than the CPG pattern or the heartbeat rhythm (Figure 1D). This result is somewhat surprising, and the implications are not yet understood, but it suggests that the properties of the motor neurons have a greater influence on the consistency of phase relationships, and hence the output, than the pacemakers do (Wright and Calabrese, 2011).

Previous research had already suggested that the variability between comparable CPG components affects the possible output of a network (Ransdell et al., 2013; Lane et al., 2016). The work of Wenning et al. takes this further by showing that the differences between CPG components contribute to the variability in an entire population of leeches. Moreover, the variability of the CPG output does not change at each level. Different life histories or genetics undoubtedly account for some of the observed differences, but this work provides a roadmap to determine how CPG variations may also affect inbred or simplified ‘synthetic’ populations.
